# Use and timing of referral to specialized palliative care services for people with cancer: A mortality follow-back study among treating physicians in Belgium

**DOI:** 10.1371/journal.pone.0210056

**Published:** 2019-01-17

**Authors:** Gaëlle Vanbutsele, Luc Deliens, Veronique Cocquyt, Joachim Cohen, Koen Pardon, Kenneth Chambaere

**Affiliations:** 1 End-of-Life Care Research group, Vrije Universiteit Brussel (VUB) & Ghent University, Brussels, Belgium; 2 Department of Public Health and Primary Care, Ghent University, Ghent, Belgium; 3 Department of Medical Oncology, Ghent University Hospital, Ghent, Belgium; University of Alabama at Birmingham, UNITED STATES

## Abstract

**Background:**

Referral to specialized palliative care services (SPCS) occurs often late in the illness trajectory but may differ across cancer types. We examined differences between cancer types in the use and timing of referral to specialized palliative care services (SPCS) and in the reasons for non-referral.

**Methods:**

We conducted a population-based mortality follow-back survey among physicians who certified a representative sample of deaths in Flanders, Belgium. We focused only on sampled death cases of cancer (n = 2392). The questionnaire asked about the use of the existing types of SPCS and the timing of referral to these services.

**Results:**

Response rate was 58% (1394/2392). Patients who died from breast, respiratory, head and neck, genitourinary or gastrointestinal cancer had higher chances of using SPCS compared to hematologic cancer patients. The most prevalent reason for non-referral was that regular care sufficiently addressed palliative and supportive care needs (51%). This differed significantly between cancer types ranging from 77,8% for breast cancer and 42.1% for hematologic cancer. A second prevalent reason for not using SPCS was that it was not meaningful (enough) (23.9%), particularly for hematologic malignancies (35,1%) and only in 5.3% for breast cancer.

**Conclusion:**

Differences in referral across different types of cancer were found. Referral is more often delayed or not initiated for patients with hematologic cancer, possibly due to differences in illness trajectory. An influencing reason is that physicians perceive palliative care as not meaningful or not meaningful enough for these patients which may be linked to the uncertainty in the disease trajectory of hematologic malignancies.

## Introduction

Cancer patients often suffer from various disease- or treatment-related symptoms that may result in high symptom burden, physical, emotional and spiritual suffering, which reduces quality of life [1,2]. Quality of life is increasingly recognized as an important outcome for cancer patients and studies have demonstrated that it is positively influenced by the early integration of specialized palliative care services (SPCS) in standard oncology care [3–6]. The definition of palliative care (PC) of the World Health Organization (WHO) also states that it should be integrated early in the disease trajectory of patients suffering from life-limiting illnesses alongside disease modifying therapies [7]. Not every patient requires specialized palliative care since palliative care needs may be met by physicians with basic palliative care skills but in cases of complex palliative care problems, referral to specialized palliative care services (SPCS) is needed [8–10]. Advanced cancer is often associated with high and complex symptom burden but research shows low referral to specialized palliative care programs in oncology care [11]. In addition, SPCS are traditionally still initiated very close to death and not early in the disease trajectory [12–14].

Previous research of use and timing of SPCS showed that cancer patients were referred late (median: 16 days before death) in the disease trajectory but perceived cancer patients as a homogeneous group and did not examine differences in referral between cancer types [12,14]. However, different cancer types have distinct symptom clusters and illness trajectories, and physicians have reported that these aspects influenced their past decisions to refer to SPCS [15–19]. In addition, also non patient-related characteristics such as physician and staff’s knowledge about palliative care and SPCS influences referral practices, practice specialty even influences patient’s survival after referral to SPCS [18]. This suggests that differences in referral and timing of referral might exist between oncology subspecialties and hence cancer types [20].

Some studies looked at the availability of SPCS for patients in cancer types and reported significant differences between cancer types in timing of referral to palliative care [21,22]. However, this evidence is limited to data at an institutional level, not at population level. Information is needed at a population level in order to assess the actual integration of palliative care in oncology care across care settings.

The aim of this population-based retrospective study is to examine whether there are differences between cancer types in the use of SPCS, in the type of service used in particular in the timing of referral to SPCS, and in the reasons for non-referral.

## Methods

### Death certificate study

We conducted a population-based survey, based on a large and representative sample of death certificates in Flanders, Belgium. The Flemish Agency for Care and Health, the central administration authority for processing death certificates, selected a random sample of deaths in the first half of 2013. For this analysis, we focus only on sampled death cases of malignant cancer (ICD-10 C00-C97). One third of all cancer deaths of Belgian residents aged one year or older were sampled from January 1^st^ until June 30^th^ 2013. This resulted in a sample of 2,669 cancer related-deaths.

Within two months of the death, every physician certifying a death certificate in the sample received a questionnaire about the end-of-life care and decision making regarding this case. The physicians were requested to complete the questionnaire consulting the patient’s medical file. If the certifying physician was not the treating physician, the questionnaire was to be passed on to the treating physician. A one-page questionnaire was mailed to all non-responding physicians, inquiring about reasons for not participating.

### Questionnaire

The questionnaire about end-of-life care and decision making has been repeatedly used in earlier studies [23–25], the questions regarding palliative care referral, which were tested by a panel of 10 physicians via cognitive interviews to correct for ambiguities, were added to the questionnaire. The questionnaire first asked whether death had been sudden and totally unexpected. If answered negatively–and hence referral to specialised palliative care services could not be precluded–physicians were asked whether and when they had: (1) initiated specialized palliative care services, (2) what the treatment goal was in the last week of life and (3) what the reasons were for not using SPCS.

1. *Use of specialised palliative care services*. The physician was asked if one or more of the existing types of SPCS in Belgium had been involved in the care of the deceased patient. These services are: multidisciplinary palliative home care teams (team skilled in palliative care who care for the patient and support the caregivers at home), mobile hospital-based palliative care teams (multidisciplinary team that guides palliative care in the different wards of the hospital), inpatient palliative care units (separate wards in the hospital with a multidisciplinary team delivering palliative care) and a palliative care reference person in a nursing home (usually a nurse) trained in and responsible for palliative care. The physician was also asked to indicate the timing of the referral, i.e. the number of days between the first referral to a SPCS and death.
2. *Treatment goal in the last week of life*. The physician was asked to indicate the main treatment goal in the last week of life, the possibilities were life prolongation/curation or comfort/palliation.
3. *Reasons for not using palliative care services*. When none of the SPCS been involved in the end-of-life care of the patient, physicians could mark the reasons why no such services were used: 1) palliative care was not meaningful or not meaningful enough (not relevant, i.e. not of value), 2) palliative care was not available, 3) existing care already sufficiently addressed the patient’s palliative and supportive needs, 4) there was not enough time to initiate palliative care, 5) in order not to deprive the patient and/or family of hope, 6) the patient did not want it, 7) the family did not want it or 8) another reason (with the request to specify the reason in text). The reasons in this category were afterwards checked by the researchers and allocated to one of the previous categories where possible. Physicians could tick more than one reason for each patient. The possible reasons were selected based on relevant literature about factors hindering the use of palliative care services [17,18,26–29] and on preceding qualitative research on reasons for not using palliative care [30].

Demographic and clinical patient data were obtained from the death certificate and linked anonymously after data collection.

### Analysis

The response sample was corrected to be representative of all cancer deaths in the first half of 2013 in terms of age, sex, marital status, province of death, cause of death and place of death (adjustments only needed for place of death). After this weighting procedure there were no significant differences between response sample and all deaths on any of these variables. Due to the format of the questionnaire, only non-sudden deaths were considered. All analyses were based on complete cases. Pearson chi square tests analyses were performed for patient and treatment characteristics according to cancer types. For referral to SPCS and reasons for not referring to SPCS non-sudden deaths were considered due to the format of the questionnaire. We also performed a multivariable logistic regression to look at differences between cancer types in SPCS referral and to explore characteristics of cancer patients that are independently related to the use of specialised palliative care services. The non-parametric Kruskal-Wallis test was calculated to test for differences in timing of referral between cancer types. All calculations were made using the in SPSS version 23.0 (SPSS, Inc., Chicago, IL).

### Anonymity and ethical considerations

To guarantee absolute anonymity for participating physicians, a lawyer served as an intermediary between responding physicians, researchers and the Flemish Agency for Care and Health, ensuring that completed questionnaires could never be linked to a particular patient or physician. After data collection a one-page questionnaire was mailed to all non-responding physicians, inquiring about reasons for not participating. The study was approved by the Ethical Review Board of the University Hospital of the Vrije Universiteit Brussel, the Belgian National Disciplinary Board of Physicians, and the Belgian Privacy Commission.

## Results

Questionnaires were returned for 1,394 of 2,669 cancer deaths. Non-response questionnaires revealed that responding was impossible in 277 cases, for example because the physician did not have access to the medical file or the patient could not be identified. Therefore, the response rate was 58.3% (1,394 of 2,392 cases).

### Case characteristics

Patients dying from different cancer types (breast, respiratory, gastrointestinal, genitourinary, head and neck, hematologic or other cancers) differed significantly in terms of distribution for sex, age, living situation and marital status (Table 1).

**Table 1:** Patient characteristics of all non-sudden cancer deaths according to cancer types (in percentages).

	All cancer deaths (n = 1079)	Breast (n = 90)	Gastrointestinal (n = 317)	Respiratory (n = 266)	Genitourinary (n = 187)	Head and Neck (n = 31)	Hematologic (n = 89)	Other* (n = 98)	p-value†
Sex									<0.001
*Male*	57.6	0	59.9	71.1	58.8	77.4	64.0	51.5	
*Female*	42.4	100	40.1	28.9	41.2	22.6	36.0	48.5	
Age at death									<0.001
*1-64y*	25.1	34.4	21.4	31.1	10.7	58.1	18.2	36.1	
*65-79y*	41.3	41.1	43.1	46.8	43.9	29.0	29.5	29.9	
*≥ 80y*	33.6	24.4	35.5	22.1	45.5	12.9	52.3	34.0	
Place of death									0.057
*Hospital (excl*. *PCU)*	40.5	33.0	40.0	44.3	37.5	35.5	51.7	35.7	
PCU	13.2	22.0	12.4	12.5	12.5	16.1	10.1	13.3	
*Home*	34.9	28.6	36.2	37.5	34.2	41.9	25.8	36.7	
*Care Home*	10.7	16.5	10.5	5.7	14.1	6.5	12.4	13.3	
*Other*	0.7	0.0	1.0	0.0	1.6	0.0	0.0	1.0	
Living Situation									0.039
*Alone*	*21*.*5*	*20*.*2*	*18*.*5*	*19*.*7*	*26*.*1*	*25*.*8*	*29*.*2*	*19*.*6*	
*In household with others*	*67*.*5*	*62*.*9*	*72*.*7*	*72*.*7*	*60*.*3*	*64*.*5*	*55*.*1*	*68*.*0*	
*Institution*	*11*.*1*	*16*.*9*	*9*.*3*	*7*.*6*	*13*.*6*	*9*.*7*	*15*.*7*	*12*.*4*	
Marital status									<0.001
*Unmarried*	7.1	3.3	7.6	6.8	4.3	16.7	10.0	10.2	
*Married*	57.3	52.2	59.3	62.4	58.3	53.3	45.6	52.0	
*Widowed*	26.0	37.8	26.2	16.2	28.9	13.3	38.9	27.6	
*Divorced*	9.5	6.7	6.9	14.3	8.6	16.7	5.6	10.2	
*Other*	0.1	0.0	0.0	0.4	0.0	0.0	0.0	0.0	
Attending physician									0.017
*Hospital specialist*	50.0	50.0	47.8	53.9	47.1	38.7	59.6	47.4	
*Family physician*	46.6	50.0	46.8	43.8	49.7	48.4	39.3	50.5	
*Other*	3.3	0.0	5.4	2.2	3.2	12.9	1.1	2.1	

Percentages are column percentages.

^†^Pearson χ2 test testing for differences between the types of cancer: breast, respiratory, colorectal, genitourinary and other.

* Other: bone & articular cartilage -, skin-, eye; brain & central nervous system-, thyroid & endocrine glands-, ill-defined; secondary and unspecified sites and independent multiple sites. Only non-sudden deaths are reported since physicians were not asked to answer questions related to referral to specialist palliative care services when death had occurred suddenly and totally unexpectedly.

### Use of specialized palliative care services

The use of specialized palliative care services differed between cancer types (p = 0.007). People who died from head and neck cancer used specialized palliative care services the most (86.3%), in all other cancer patients over 73% get access to SPCS, except for people who died from hematologic cancer with 56.4%.

**Fig 1:**
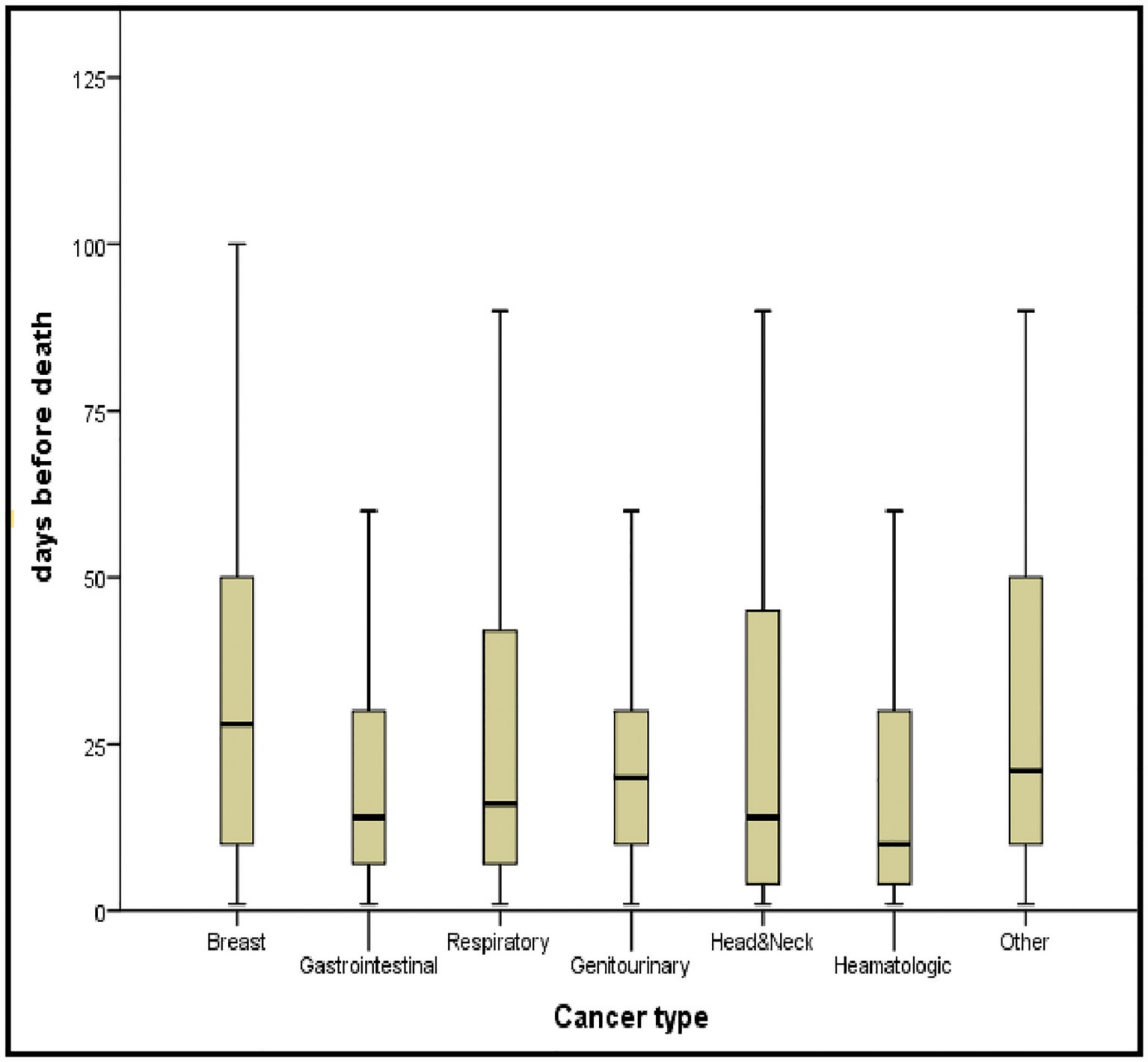
Box plot of timing of referral to palliative care services (days before death) per cancer type. Outliers are not shown and differ per cancer type: Breast: > 114 days (n = 7); Gastrointestinal: > 64 days (n = 26); Respiratory: >107 days (n = 11); Genitourinary: > 60 days (n = 14); Head &Neck: >107 days (n = 1); Hematologic: > 65 days (n = 5); Other: > 105 (n = 5).

The median timing of referral before death was highest in breast cancer patients (29 days) and lowest in hematologic cancer patients (10 days) (Fig 1). Fig 1 shows an important variation in timing of referral to specialized palliative care services in the groups of patients who died from breast cancer, respiratory cancer or head and neck cancer, while for gastrointestinal, genitourinary and hematologic cancer this within-group variation was less. However, these differences between cancer types were not statistically significant (Table 2). The treatment goal in the last week of life differed between cancer types (p = > 0.044). However, considering multiple testing, the evidence of differences between cancer types is not strong enough to make any clear conclusions.

**Table 2:** Rates, timing of referral to specialist palliative care services and treatment goal in the last week of life; % of non-sudden deaths.

	All cancer deaths (n = 1079)	Breast (n = 90)	Gastro-intestinal (n = 317)	Respiratory (n = 266)	Genitourinary (n = 187)	Head and Neck (n = 31)	Hematologic (n = 89)	Other (n = 98)	p-value
Any type of PC†	74.2	79.5	73.1	75.9	75.6	86.3	56.4	78.4	0.007
Palliative care support at home	30.8	30.9	29.3	35.1	27.8	43.8	20.2	35.1	0.076
Hospital-based palliative care service (excl. Palliative care unit)	32.1	32.2	32.5	36.3	31.3	37.2	27.7	23.5	0.503
Palliative care unit	15.3	19.7	15.8	14.0	15.2	23.8	12.8	15.0	0.805
Palliative care reference person in a nursing home	3.6	13.7	5.7	1.6	9.0	5.0	3.5	9.5	<0.001
Other	3.1	3.5	3.4	2.6	5.6	0.0	0.0	1.8	0.172
Median days prior to death*‡	16	29	14	19	20	14	10	21	0.173
P25 & P75	7; 33	10;52	7; 30	7; 47	10;30	4; 45	3; 28	10; 48	
Mean	37	44	35	39	36	37	31	35	
Treatment goal in the last week									0.044
*Life prolongation/curation*	30.3	25.2	33.4	26.4	29.2	23.1	41.1	30.2	
*Comfort/palliation*	69.7	74.8	66.6	73.6	70.8	76.9	58.9	69.8	

Percentages are column percentages. Percentages may add up to more than the total percentage of referrals because more than one palliative care service was used in some cases.

*Calculations for only patients with a referral to palliative care services, n = 792. Missing values 12.8% (n = 101)

^†^Pearson *χ*^2^ test testing for differences in referral between the seven different cancer types.

^**‡**^Kruskal-Wallis test testing for differences in time of onset between the seven cancer types, minimum 1, maximum 666

### Characteristics associated with using specialized palliative care services

When comparing the use of palliative care services between solid cancer types and hematologic cancer in multivariable logistic regression, and after bringing socio-demographic characteristics into consideration, patients who died from breast, gastrointestinal, respiratory, head and neck, genitourinary cancer and patients dying from other cancers had higher odds of using specialized palliative care services compared to patients dying from hematologic cancer (Table 3).

**Table 3:** Multivariabele odds ratios and probabilities for referral to specialist palliative care services, according to type of cancer and sociodemographic characteristics for all non-sudden cancer deaths.

	Using SPCS* OR (95% CI) multivariate	Model-estimated Probabilities (%)
Type of Cancer		
*Breast*	***2*.*25 (1*.*06–4*.*80)***	***0*.*76 (0*.*64–0*.*85)***
*Gastrointestinal*	***2*.*00 (1*.*17–3*.*44)***	***0*.*74(0*.*68–0*.*79))***
*Respiratory*	***2*.*29 (1*.*31–4*.*02)***	***0*.*74 (0*.*68–0*.*80)***
*Genitourinary*	***2*.*28 (1*.*25–4*.*15)***	***0*.*75 (0*.*67–0*.*81)***
*Head & Neck*	***4*.*58 (1*.*34–15*.*65)***	***0*.*87 (0*.*70–0*.*95)***
*Hematologic*	Ref Cat	0.59 (0.47–0.69)
*Other*	**2.60 (1.28–5.27)**	**0.81 (0.71–0.88)**
Living situation		
*Alone*	*1*.*22 (0*.*80–1*.*85)*	0.79 (0.73–0.85)
*In household with other*	Ref Cat	0.77 (0.72–0.81)
*Institution*	*0*.*72 (0*.*46–1*.*11)*	0.71 (0.62–0.79)
Age		
*18-64y*	*1*.*32 (0*.*86–2*.*04)*	*0*.*78 (0*.*71–0*.*83)*
*65–79 y*	**1.46 (1.01–2.10)**	**0.79 (0.74–0.84)**
*≥ 80 y*	Ref Cat	0.70 (0.64–0.75)
Sex		
*Male*	Ref Cat	0.73 (0.67–0.78)
*Female*	***1*.*42 (1*.*01–1*.*99)***	***0*.*78 (0*.*73–0*.*83)***

The test of model effects was significant (p = 0.02) indicating that our model has a significant model estimation fit. Cox & Snell R Square (0.032) and Nagelkerke R Square (0.047) indicate that our model has modest predictive power.

### Reasons for not using palliative care services

Of the 19% (n = 268) of cancer patients who were not referred to SPCS, the physician perceived the regular care as sufficiently addressing the patient’s palliative and supportive needs in 51% of the cases (Table 4). This differed significantly between cancer types, ranging from 77.8% in breast cancer (77,8%) to 42.1% in hematologic cancers (42.1%), without taking head and neck cancers into account (n = 3). Only 4.6% of patients who died of breast cancer did receive neither palliative care nor regular care which, according to the physician, sufficiently met palliative and supportive care needs. This ranged between 15.1% to 8.7% for all other solid cancer types. Patients with hematologic cancer had the highest percentage (25.2%) (Not shown in table).

**Table 4:** Reasons given by physicians for not using specialist palliative care services (PCS) in patients who died of cancer.

	Care sufficient (%)	Not meaningful (%)	Not enough time (%)	Patient did not want (%)	Family did not want (%)	Not available (%)	Not take away hope (%)
All cancer types (n = 268)	**51.3**	**23.9**	26.4	**12.7**	5.2	1.1	0.7
Cancer type							
*Breast (n = 19)*	**77.8**	**5.3**	16.7	**10.5**	0.0	0.0	0.0
*Gastrointestinal (n = 83)*	**44.0**	**25.3**	27.7	**15.7**	3.6	2.4	0.0
*Respiratory (n = 62)*	**52.4**	**22.6**	23.8	**15.6**	11.1	0.0	1.6
*Genitourinary (n = 45)*	**64.3**	**13.3**	21.4	**4.8**	7.0	2.3	2.2
*Head and Neck (n = 3)*	**0.0**	**33.3**	0.0	**66.7**	0.0	0.0	0.0
*Hematologic(n = 37)*	**42.1**	**35.1**	32.4	**10.5**	0.0	0.0	0.0
*Other (n = 19)*	**52.6**	**42.1**	42.1	**5.3**	5.0	0.0	0.0

Full response answers which physicians could indicate as a reason for not using palliative care services were respectively: the care already sufficiently addressed the patient’s palliative and supportive needs; palliative care was not meaningful or not meaningful enough; there was not enough time to initiate palliative care; patient did not want it; family did not want it; palliative care was not available; to not take away the hope of the patient and/or the family.

Abbreviations: PCS = palliative care services.

Percentages are row percentages. Percentages may add up to more than 100 because more than one reason could be indicated in some cases.

*Bivariate Pearson *χ*^2^ test testing for differences in between cancer types. Bold denotes significant at p < .05.

Another prevalent reason, differing significantly between cancer types, was that palliative care was not meaningful or not meaningful enough according to the physician (23.9%), particularly in hematologic cancer (35.1%). Interestingly, this reason was only mentioned in 5.3% of breast cancer patients. According to the physicians, not having enough time to refer the patient to palliative care services also occurred frequently (26.4%) but this did not differ significantly between cancer types. In some cases (12.7%) patients refused to be referred to specialized palliative care services which differed significantly between cancer types.

## Discussion

Our study found that an important proportion of Flemish cancer patients were referred to specialized palliative care services prior to death, varying from 56% for hematologic cancer to 75% for breast cancer. However, referral mostly occurred late in the disease trajectory and chances for referral are not equal for patients dying from various cancer types. Patients with hematologic cancer have smaller chances of referral to any SPCS compared to all other solid cancers, even after controlling for patient characteristics. Reasons for non-referral to SPCS also differed significantly between cancer types. The most indicated reasons were that 1) standard care already sufficiently addressed the patient’s palliative care needs, 2) there was not enough time and that 3) palliative care was not meaningful or not meaningful enough.

This study has several strengths. This population-based study constitutes a large representative sample of cancer deaths in Flanders and has an appropriate design to evaluate the referral or non-referral to specialized palliative care services of deceased cancer patients. This study is not limited with regard to care setting or sample size. Furthermore, because of the nationwide scope of this study, our results allow for international comparative research. A shortcoming of this study is the one- to two-month interval between death of the patient and the sending of the questionnaire which might result in recall bias for some cases [24,31,32]. Additionally, treating physicians might not always have knowledge about the specific palliative care services provided across different setting of care (e.g. community versus hospital) resulting in a possible underestimation of the use of specific specialized palliative care services. We also did not exclude patients under the age of 18 who may have different care compared to adults. However, we believe this to have little influence on our findings due to the small N of child and adolescent deaths in our sample. Further, the questionnaire is necessarily (such as all surveys) a reduction of reality and cannot capture all the nuances of care. Our study also focuses only on formal care of SPCS, the palliative care approach provided by other health care provisional is neglected. Further, we did not correct for multiple testing in our analysis and due to width of the 95% confidence interval and the small sample size of the head & neck patients in our study, no clear conclusion can be drawn for this group of patients regarding the odd of referral to SPCS, compared to hematology patients. Last, the reasons for not using palliative care only include aspects related to treatment options and are based on the physician’s perspectives. Perspectives of patients, carers and other professional caregivers is missing.

Our findings show that SPCS are widely available for cancer patients in Belgium which is similar to other countries such as Canada and Australia[33,34], In addition, most of the cancer patients in this study were either referred to one or more of the different services before death or received regular care which sufficiently supported the palliative care needs according to the physician. These are reassuring results indicating that a palliative care approach is not neglected in oncology care and concur with the palliative care model in Belgium which is very much based on specialized palliative care provided by specialized palliative care professionals and less on a generalist palliative care approach [35].

The idea of palliative care integrated early in cancer care has gained momentum in recent years[36], yet in day-to-day practice in Belgium specialized services are only involved in the final days of life especially compared to other countries. In our study timing of referral to SPCS did not differ significantly between cancer types, but was the longest for breast cancer patients (median of 29 days) and the shortest for patients with hematologic cancers (median of 10 days). This is late compared to other countries such as the United Kingdom (UK) where patients with breast cancer and patients with hematologic cancer were referred 43.5 and 26 days prior to death respectively[37]. In addition, institutional data shows that in the United States, and in Australia cancer patients were referred, respectively 42 days and 54 days prior to death [21,38]. The Belgian health care system is likely to have an impact on our findings since the current reimbursement and eligibility criteria to be granted a “palliative status’, which covers the costs of additional medicines and materials through health insurance, is that patients are expected to live three months or less. This might strengthen the idea of palliative care as terminal care. This late referral is an important concern since the end-of-life care in Belgium is more hospital centric with high chemotherapy utilization, compared to other countries such as the United States [39]. This raises the important issue whether palliative care professionals in Belgium have enough time to establish comfort care for cancer patients at the end-of-life.

The found discrepancies in use of specialized PC services between hematologic cancer (54%) and solid cancer types (78%) concur with findings from the UK (12% versus 28%) and Canada (25% versus 36%) [40]. An important finding of our study was that 25% of patients suffering from hematologic cancer were not referred to SPCS or did, according to the physician, not receive usual care that sufficiently addressed the palliative care needs while this is only the case for 5% of patients who died of breast cancer and about 12% for patients who died from all other types of cancer. Several reasons might be related to the discrepancies. One suggested reason is that patients suffering from advanced cancer of solid tumors experience higher, more complex and more diverse symptom burden and thus clearer indications of the need for referral to SPCS [21,41]. Another reason is that the illness trajectory for hematologic cancer is less predictable. Hematologic cancer is often characterized by acute exacerbations of the illness followed by highly technical therapies that can continue over many years. This, along with a rapid dying trajectory, results in difficulties in assessing the right time to refer to SPCS [19,42,43].

Nonetheless, hematological cancer patients have been shown to have similar symptom control needs to other cancer patients in all phases of the disease and evidence suggests that hematologic cancer patients have similar patterns of physical decline at the end of life [22, 44–46]This suggests that other factors than clinical and treatment characteristics might also play a role in referral to SPCS. Organizational aspects might influence referral practices since transfusion of blood products might benefit the quality of life of hematologic cancer patients but is often impossible in a palliative setting [47] Also intrapersonal aspects, such as attitudes and perceptions of patients, family and medical staff toward PC may affect referral to SPCS. Interestingly, we found that physicians frequently perceive palliative care as not meaningful or not meaningful enough, particularly so for patients who died from hematologic cancer. Possible hypotheses for these findings might be that Belgian hematologic oncologists, compared to solid tumor oncologists, more frequently perceive palliative care as end-of-life care and perhaps also as antithetical to cancer care [48]. Research shows that hematologists often view treatment goals or disease characteristics in hematology care as incompatible with palliative care [49–52]. On the other hand, it is also plausible that hematologists consider the delivery of palliative care to be integral to their role, hematologists might be concerned that referral to SPCS may alarm the patient and family caregiver[53,54]. Research has reported that the close relationship developed between hematologists and their patients during the treatment course might be a barrier for collaboration between both disciplines[55].

Our results also point out that palliative care is very well integrated in the usual care for breast cancer patients since 95% of patients who died of breast cancer were referred to SPCS or received usual care that sufficiently addressed the palliative care needs according to the physicians. The mechanism behind these differing results related to cancer type remain unclear. For breast cancer, for example, psychosocial interventions influencing quality of life are well integrated in the regular care which might lead to higher awareness of the benefits of palliative care among professional health carers and patients, resulting in earlier referral to palliative care [56]. Similarly, research shows that the dichotomized thinking of some hematologists, with the “either/or” approach to active treatment and palliative care may act as a barrier for collaboration with SPC[55]. This might influence patients and families who as a result underestimate the benefits of palliative care and continue to perceive palliative care as terminal care only. Evidence shows that bereaved family members of patients with hematologic cancer felt unprepared for the dying experience and felt unsupported to deal with spiritual pain. This evidence supports the missed opportunity of the benefit of a palliative care approach [44,54].

## Conclusion

Despite increasing evidence of the benefit of early integration of palliative care for cancer patients, our study contributes to the awareness that the use of palliative care services in Belgium is high but still occurs late in the disease trajectory. Palliative care is still perceived as terminal care which may lead to unmet palliative care needs of cancer patients and their families, particularly in hematologic malignancy. For these and other cancer patients, further research is needed to test the beneficial effects of early integration of palliative care in oncology care.
